# IL-10 and IL-22 in Mucosal Immunity: Driving Protection and Pathology

**DOI:** 10.3389/fimmu.2020.01315

**Published:** 2020-06-26

**Authors:** Hua-Xing Wei, Baolong Wang, Bofeng Li

**Affiliations:** ^1^Division of Life Sciences and Medicine, Department of Laboratory Medicine, The First Affiliated Hospital of USTC, University of Science and Technology of China, Hefei, China; ^2^Division of Life Sciences and Medicine, Department of Medical Oncology, The First Affiliated Hospital of USTC, University of Science and Technology of China, Hefei, China

**Keywords:** IL-10, IL-22, IBD, colorectal cancer, ILC, inflammation

## Abstract

The barrier surfaces of the gastrointestinal tract are in constant contact with various microorganisms. Cytokines orchestrate the mucosal adaptive and innate immune cells in the defense against pathogens. IL-10 and IL-22 are the best studied members of the IL-10 family and play essential roles in maintaining mucosal homeostasis. IL-10 serves as an important regulator in preventing pro-inflammatory responses while IL-22 plays a protective role in tissue damage and contributes to pathology in certain settings. In this review, we focus on these two cytokines in the development of gastrointestinal diseases, including inflammatory bowel diseases (IBD) and colitis-associated cancer (CAC). We summarize the recent studies and try to gain a better understanding on how they regulate immune responses to maintain equilibrium under inflammatory conditions.

## Introduction

The IL-10-related cytokine family includes several members, IL-10, IL-19, IL-20, IL-22, IL-24, IL-26, IL-28A/B, and IL-29, which also belong to the class 2α-helical cytokines (1). IL-19, IL-20, IL-22, IL-24, and IL-26 also belong to the IL-20 sub-family. All members of the IL-10 cytokine family signal through heterodimeric receptors composed of an α-chain and a β-chain. Although the IL-22 sequence shows only 22% homology with the sequence of IL-10, the protein structure of IL-22 is remarkably similar to that of IL-10 (2). IL-10, IL-22, IL-26, IL-28, and IL-29 share common usage of IL-10Rβ, while IL-10Rα specifically binds to IL-10. Because IL-22Rα is also used by IL-20, IL-22, and IL-24, it is difficult to draw conclusions about IL-22's function using knockout mice for its receptors.

The best-characterized cytokines of the IL-10 cytokine family are IL-10 and IL-22. IL-10 is regarded as the most important cytokine for suppressing pro-inflammatory responses in the immune system. IL-22 is believed to act exclusively on epithelial cells to promote cell regeneration and tissue repair. Particularly at the intestinal barrier, IL-10 and IL-22 play essential roles in the prevention or induction of mucosal damage and the development of colitis-associated cancer (CAC) (3, 4), which will be reviewed here as a major topic.

## Overview of IL-10 and IL-22

IL-10 exerts its protective functions by regulating over-exuberant immune responses and autoimmune pathologies (5). Thirty years ago, Th2 cells were found to produce a factor that inhibited Th1 cell function (6); this factor was later named IL-10 (7). The human IL-10 gene, present on chromosome 1q32, is 4.7 kb long and includes 5 exons. The murine IL-10 locus spans 5.1 kb on chromosome 1E4. The sequence identity and transcription binding sites between human and murine IL-10 are conserved (8). IL-10 not only prevents cytokine production, but also the expression of chemokine (9) and co-stimulatory molecules (CD80, CD86, and MHC Class II) (10). IL-10 binds to IL-10Rα and IL-10Rβ, which are commonly expressed on most immune cells. Therefore, IL-10 can regulate different innate and adaptive immune cells to evade the development of immune pathologies in different ways, such as inducing Treg and Tr1 cells or displaying an autocrine inhibitory effect on macrophages and dendritic cells (DCs) (11).

IL-22 was first identified by Renauld et al. in IL-9-stimulated murine T cells (12). Murine IL-22 gene is localized on chromosome 10 while human IL-22 gene is located on chromosome 12q15, near the genes that encode for IFN-γ and IL-26, other members of the IL-10 family (13). The IL-22 gene includes five exons and a 537 bp-long open reading frame that encodes for a 179 amino acid protein. Mouse and human IL-22 share 79% homology (14). Because the IL-22 receptor is widely expressed on epithelial cells located at boundary tissues, such as gut, lung, liver, and skin, the major function of IL-22 is to provide a protective response against pathogens at barrier surfaces (15, 16). The IL-22/IL-22R pathway has been shown to modulate the expression of many genes involved in tissue protection, survival, differentiation, and remodeling (17–19). Although IL-22 is mainly produced by the lymphoid lineage cells, including Th17 cells, γδ T cells, natural killer T cells, and innate lymphoid cells (ILCs), it has been reported that myeloid cells (macrophages and neutrophils) also produce IL-22 (20, 21).

**Figure 1:**
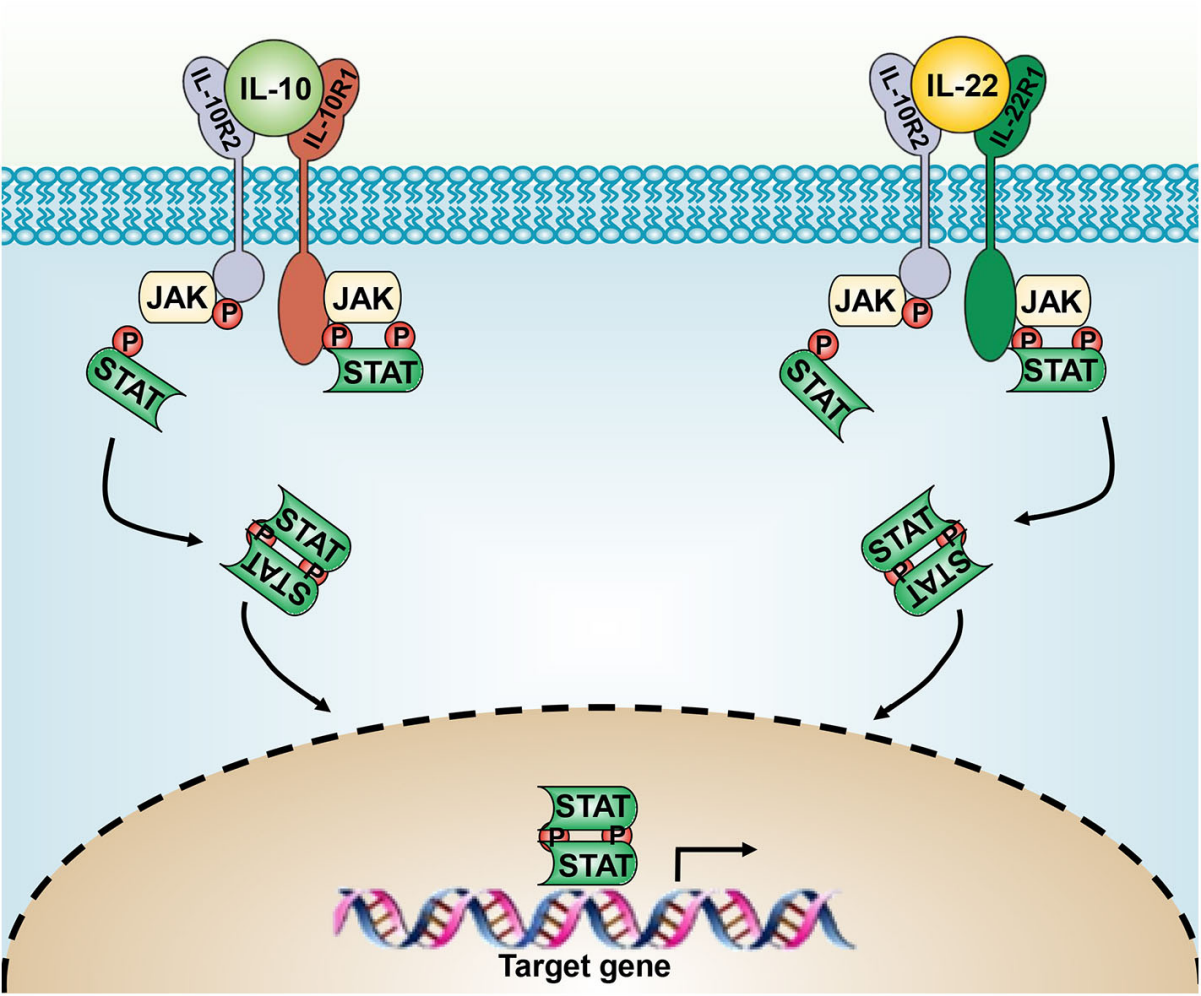
The IL-10 and IL-22 signaling pathways. IL-10 and IL-20 signal through the heterodimeric receptor complexes IL-10Rα/IL-10Rβ and IL-22Rα/IL-10Rβ, respectively. JAK/STAT-3 signaling leads to the expression of downstream genes.

IL-22 has two heterodimeric transmembrane receptors, IL-22R1 and IL-10R2, which subsequently activate the JAK/STAT3, ERK, and JNK pathways (22). Similarly, IL-10 also drives its expression through the JAK-STAT signaling pathway (23) (Figure 1). On translocation to the nucleus, Stat dimer drives the transcription of Stat3-responsive genes, including SOCS-1 and SOCS-3 (24), thus mediating the anti-inflammatory activities of IL-10. IL-22-induced STAT3 activation in epithelial cells enhances their regeneration during tissue damage. Although STAT3 activation is responsible for most of the physiological responses of IL-10 and IL-22, both of them can activate STAT-1 and STAT-5 under certain conditions (2, 25).

## Regulation of IL-10 and IL-22 Secretion by Mucosal Immune Cells

The intestinal flora, largely composed of resident bacteria, is most densely populated in the GI tract. Tolerance of the endogenous microbe is advantageous to the host while inappropriate immune responses are normally controlled by the innate and adaptive immune systems. IL-10 can be produced by various immune cells, including DCs, macrophages, mast cells, natural killer (NK) cells, eosinophils, neutrophils, CD4^+^ and CD8^+^ T cells, and B cells (5, 26, 27). IL-10 production was first described in Th2 cells (6), following with Th1, Th9, and Th17 cells were also shown to produce IL-10 (5). CD4^+^Foxp3^+^ regulatory T cells and IL-10-producing T (Tr-1) cells have non-redundant functions in controlling intestinal inflammation (26, 28). In addition to T helper cells, macrophages, DCs, and neutrophils are also known to secrete IL-10 (29).

Several transcription factors, including Blimp-1, c-Maf, and GATA3, serve as potential regulators of IL-10 expression. Blimp-1^−/−^ mice have been shown to develop a lethal multiorgan inflammatory disease caused by an accumulation of effector and memory T cells (30). Inactivation of c-MAF in the Treg cells has been found to result in dysfunction of IL-10 production and such mice were prone to spontaneous colitis (31). The transcription factor GATA3 has been described as a master regulator of IL-10 expression (32). In addition, CCAAT/enhancer binding protein-β (C/eBPβ) (33) and NF-κB p50 (34) are able to bind to the IL-10 promoter, activating IL-10 transcription in macrophages.

At the mucosal sites, IL-22 is mainly produced by the lymphoid lineage cells, including CD4^+^ and CD8^+^ T cells, γδ T cells, NK cells, and ILCs (35, 36). Th1 cells were found as a source of IL-22, initially (37). Although Th17 cells can produce both IL-22 and IL-17, it is noteworthy that a distinct human CD4^+^ T cell subset, known as Th22 cells, only produces IL-22 but not IL-17 (17, 38). Th22 cells produce their own cytokine profiles, such as IL-22, IL-26, and IL-33, which can be stimulated by IL-6, IL-21, and IL-23 (39); however, there is a debate about whether the Th22 cells are derived from Th17 cells. Apart from the adaptive cells, innate cells, including the ILCs and lymphoid tissue inducer (LTi) cells also serve as important sources of IL-22, particularly in the gastrointestinal tract (40). Th17 cells, type 3 innate lymphoid cells (ILC3s), and LTi cells express CCR6 and IL-23R (41). The Lymphotoxin (LT) pathway is only necessary for LTi cells but not for Nkp46^+^ ILC3s (42). Contrarily, loss in *Ahr* expression does not impact LTi cells formation and accumulation in the fetal intestine but influences the maintenance of gut ILC3s and Th22 cells (43, 44). Besides, IL-22-producing neutrophils have been reported to crosstalk with colonic epithelial cells to upregulate the antimicrobial peptides, RegIIIβ and S100A8 (21).

Numerous factors can regulate IL-22. IL-23 is believed to be a major inducer of IL-22 production due to the similar phenotype displayed by IL-23Rα^−/−^ mice and IL-22^−/−^ mice (45). DCs and CX3CR1^+^ macrophages are potent sources of IL-23 (46, 47), as well as IL-1β (48). Constant IL-1β signaling is required for sustained IL-22 production (49). Although IL-7 does not directly induce IL-22, it can stabilize RORγt expression in all IL-22-producing subsets (50). AhR is critical for ILC3-derived IL-22 production, because CD4^+^ T cells from AhR^−/−^ mice develop Th17 cell responses, but fail to produce IL-22 (51). Notch signaling is also necessary for both NCR^+^ and NCR^−^ ILC3 subsets to produce IL-22 (52). On the contrary, TGF-β (53), ICOS (54), and IL-27 (55) have also been shown to prevent the production of IL-22. Lastly, IL-22BP is a natural inhibitor of IL-22, having more than a 20-fold higher affinity for IL-22 than the cell surface receptor chain IL-22R1 (56), thus playing a pathogenic role in inflammatory bowel disease (IBD) (57) and multiple sclerosis (58) patients.

## IL-10 and IL-22 in Mucosal Inflammation

IL-10 and IL-22 target vastly diverse cell types and induce different downstream pathways. IL-10 prevents inflammatory responses by acting on Treg cells or macrophages, while IL-22 directly promotes tissue epithelial cell regeneration and repair. In these ways, they maintain barrier integrity and reduce tissue damage.

### IL-10

IL-10 is regarded as a major anti-inflammatory cytokine associated with many autoimmune diseases in humans and mice (2). IL-10 is highly relevant to IBD, as exhibited by the development of spontaneous enterocolitis in both IL-10^−/−^ and IL-10Rβ^−/−^ mice (59, 60). In humans, polymorphisms in IL-10 (61), IL-10Rα, and IL-10Rβ (62) have been found to be correlated with very early-onset of colitis. Genome-wide association studies (GWAS) have further revealed an important role of the IL-10 axis in IBD pathogenesis (63). All the above data indicate that IL-10 signaling is important for maintaining gastrointestinal homeostasis. Interestingly, germ-free IL-10^−/−^ mice do not develop colitis and the administration of antibiotics prevents colitis (64), indicating that the gut microbiota is necessary for the development of colitis. A single species, *Helicobacter hepaticus*, is responsible for this exacerbated disease (65). *H. hepaticus*-infected IL-10^−/−^ mice display significantly increased production of IL-12 and IFN-γ, indicating that IL-10 stimulation in response to intestinal flora is important for preventing IBD.

IL-10's protective function has been identified in many colitis models, including the DSS-induced colitis model and the CD45RB^hi^ T cell transfer colitis model, which mimic ulcerative colitis (UC) and Crohn's disease, respectively (66). IL-10 inhibits IFN-γ production by Th1 cells in mice reconstituted with CD45RB^hi^CD4^+^ T cells (67) and reduces Th17 responses in the DSS model (68, 69). As microbiota is involved in the normal physiological status of the colon (70), the immunomodulatory effects of microbiota-produced short-chain fatty acids (SCFAs) have been examined. SCFAs not only promote IL-10 production by Th17 cells and reprogram their metabolic activity toward elevated glucose oxidation (71), but also induce antigen-specific IL-10 secretion by Th1 cells to maintain the intestinal homeostasis through G-protein coupled receptor 43 (72).

Because the IL-10Rα receptor is widely expressed on T cells, B cells, macrophages, and DCs in the colon, identifying the specific immune cell lineage responding to IL-10 is important to understand IL-10's pivotal role in regulating colonic inflammation. Mice with Treg cells specifically lacking IL-10 or IL-10Rα have been found to be prone to develop spontaneous colitis (73, 74), indicating that IL-10 enables Treg cells to suppress pathogenic Th17 cell responses in colitis (75), similar to the observation in mice with Treg-specific ablation of Stat3 (76). By using conditional knockout mice with macrophages specifically lacking IL-10Rα, we found these highly activated macrophages could produce large amounts of IL-1β together with IL-6, promoting further Th17 cells development and colitis pathogenesis (77). We further identified IL-10 as a secreted inflammasome-tolerizing factor that could suppress caspase-1 activation and caspase-1-dependent maturation from pro-IL-1β to IL-1β through regulation of caspase-8 activation (78). Similarly, mice with CX3CR1^+^ macrophage-selective deletion of IL-10Rα have been found to develop severe spontaneous colitis (79). In addition, macrophages in Rag2^−/−^IL-10Rβ^−/−^ mice showed impaired iTreg generation and Treg function. Since the shared usage of IL-10Rβ by IL-10 and IL-22 is well-known (80), further studies need to investigate the separate role of these two cytokines. Clinically, the therapeutic effect of anti-TNF treatment in IBD patients is also dependent on the IL-10 signaling in macrophages (81). In the DSS-induced colitis model, IL-10 exerts its protective role through the macrophage-ROS-NO axis; lamina propria macrophages produce substantially greater levels of NO and ROS when they are unable to respond to IL-10 (82). IL-1β mediates IBD in patients with IL-10 receptor deficiency was reported thereafter (83). Taken together, these studies prove that IL-10 signaling in the intestinal macrophages is indispensable for controlling mucosal inflammation.

It is well-proven that IL-10 is required for intestinal homeostasis; despite this, the downstream signaling pathways and the molecular basis involved have not been fully examined. Recently, several pathways related to IL-10 have been identified. Lp et al. revealed that IL-10 alters macrophage function by promoting the clearance of damaged mitochondria and modulating cellular metabolism in an STAT3-DDIT4-dependent manner (84, 85). Shp2 has been found to disrupt IL-10-induced STAT3 activation and its dependent anti-inflammatory response in human and murine macrophages (86). Inhibition of GSK3β results in tolerogenic bone marrow-derived DCs with profoundly decreased C/EBPβ and CREB DNA binding activities, which leads to a reduction of IL-10 and an increase in IL-12p70 production (87). This is consistent with another observation that GSK3β deletion in CD4^+^ T cells improves the survival of T cells and ameliorates colitis (88). In T cells, IL-10 can directly restrict the activation and function of CD8^+^ T cells by inducing the expression of Mgat5 and modifying the N-glycan branching on surface glycoproteins (89). This mechanism is seen under inflammatory conditions as well; MGAT5^−/−^ mice have been found to exhibit increased susceptibility to early-onset colitis, while reduced branched N-glycosylation on mucosal T cells has been observed in case of UC patients (90). The involvement of IL-10 in this process needs to be further examined.

### IL-22

Unlike IL-10, which targets hematopoietic cells, the major impact of IL-22 is on non-hematopoietic epithelial cells and stromal cells (4), due to the restricted IL-22R expression in these cells. IL-22 can promote proliferation and barrier function. Similar to IL-10, IL-22 exerts a protective effect on mucosal inflammation in most animal models, but plays a harmful role in the anti-CD40-induced colitis model, which will be discussed later in this section.

Elevated IL-22 levels have been detected in patients with Crohn's disease (91), as well as increased IL-22 and IL-22Rα expressions in colon biopsy samples from UC patients (92). Also, in DSS-induced colitis, IL-22 has been shown to ameliorate the histological score (93) in an STAT3-dependent manner. Furthermore, STAT3 activation in epithelial cells is dependent on IL-22 rather than IL-6 (94), suggesting that targeting the STAT3 signaling pathway in intestinal epithelial cells (IECs) is a promising therapeutic approach for IBD patients (95). Initially, it was believed that IL-22-expressing NK cells are responsible for protection against intestinal injury (96). Later, these cells were identified to display a “CD3^−^CD127^+^CD56^+^NKp44^+^” phenotype (97) and were named as ILC3s (98). ILCs are innate immune cells that lack antigen specificity, enriched at mucosal surfaces, and regulate immune responses as well as tissue homeostasis (98–101). ILCs can be divided into subsets that are characterized by their production of cytokines, including ILC1 (IFN-γ) (102), ILC2 (IL-5/IL-13), ILC3 (IL-17/IL-22), and ILCreg (IL-10/TGF-β) (103).

IL-22-producing ILC3s have been shown to play a protective role in a mouse model of infectious colitis induced by *Citrobacter rodentium*. These RORγt^+^NKp46^+^ ILC3s, but not NCR^−^ ILC3s, are regulated by an intrinsic TCF-1 pathway that plays a critical role in the host defense against *C. rodentium* infection (104). Notably, Giacomin et al. found that IKKα^Δ*IEC*^ mice displayed impaired IL-22 production by RORγt^+^ ILC3s, while rIL-22 administration or transferring WT cells protected IKKα^Δ*IEC*^ mice from *C. rodentium*-induced morbidity (105). Lamina propria CX_3_CR1^+^ mononuclear phagocytes are stronger inducers of ILC3 production of IL-22 (106).

With respect to T cell-induced colitis, no significant difference has been reported between RAG1^−/−^ mice that received CD45RB^high^IL-22^+/+^ or CD45RB^high^IL-22^−/−^ CD4^+^ T cells, indicating that the mice rely on a host-derived source of IL-22 during IBD development (96). Upon CD45RB^hi^ T cell transfer, the disease severity in *Rag1*^−/−^*Ahr*^−/−^ mice has been found to be substantially higher than that in *Rag1*^−/−^ mice. *Ahr*^−/−^ mice with reduced ILC3-produced IL-22 are prone to spontaneous colitis accompanied with increased segmented filamentous bacteria and Th17 cells (107). Interestingly, TNF-like ligand 1 A (TL1A) and death receptor 3 (DR3) are a ligand–receptor pair involved in the pathogenesis of IBD. TL1A potently enhances IL-23- and IL-1β-induced production of IL-22 and GM-CSF by ILC3 (106, 108). Together with α-DR3 treatment, it causes a reduction in the ILC3 numbers in the large intestine (109).

Although widely considered as an anti-inflammatory cytokine, IL-22 plays a pathogenic role in the innate colitis model. ILC3s have been identified as the only mediator for disease induction in the anti-CD40-induced colitis model (110). Adoptively transferring CD45^+^Lin^−^Thy1^+^CD127^+^NKp46^+^ ILC3s from Rag1^−/−^ mice into NSG mice has been shown to cause severe colitis, proving that NKp46^+^ ILC3s alone are sufficient to induce innate immune colitis (111). Neutralization of IL-22 results in a significant reduction in the weight loss and colitis scores caused by the anti-CD40 injection (111), while IL-22 administration has been shown to drive more severe mucosal damage (112). Identical results were reported in another CD45RB^lo^ memory T cell transfer colitis model (113). Interestingly, transferred Treg cells reduce the ILC3 production of IL-22 through suppression of the CX3CR1^+^ macrophage production of IL-23 and IL-1β (114), indicating a potential network between adaptive and innate immune responses.

Together, such conflicting reports on the role of IL-22 in different colitis models reflect the complex function of ILC3 in relation to gut inflammation. Since it is unclear why ILC3-induced IL-22 functions as a double-edged sword and displays both pro-inflammatory and anti-inflammatory properties in maintaining gut homeostasis, particularly in case of innate cell-induced inflammation, more extensive studies are required in the future to unravel its mechanism of action.

## IL-10 and IL-22 in Colorectal Cancer

### IL-10

IL-10 has a paradoxical role in cancer development (115). IL-10 and TGF-β are considered as the two most important immunosuppressive cytokines in the immune system as they can help tumors escape immune surveillance in the tumor microenvironment. Clinical analysis shows poor prognosis of melanoma patients with high levels of IL-10 in the serum (116) and tumor tissue (117). Mechanistically, IL-10 not only inhibits MHC class II expression on APCs, but also reduces the cytotoxicity of NK cells and CD8^+^ T cells (3). Notably, while IL-10^+^ Treg cells promote tumor growth and induce T cell exhaustion, deletion of IL-10 in Treg cells has been shown to cause a drastic reduction in the expression of PD-1, TIM3, and LAG3 in intra-tumoral CD8^+^ T cells (118).

Chronic inflammation is one of the hallmarks of cancer. In the setting of intestinal inflammation, the mucosal immune response leads to colorectal cancer, as exhibited by the increased incidence of colitis-associated colon cancers in IBD patients. Sixty percent IL-10^−/−^ mice develop colorectal cancer; these mice have significantly increased levels of pro-inflammatory cytokines (IFN-γ, TNF-α, IL-1β, and IL-6), indicating the chronic intestinal inflammation related with the tumor growth (119, 120). It is interesting to note that apart from COX2 (121) and PTEN (122), which have been found to facilitate the progression of cancer in IL-10^−/−^ mice, the development of colitis also depends on IL-22, as exhibited by the elevated levels of IL-22^+^ Th17 cells in the colon of IL-10^−/−^ mice (123).

In fact, elevated levels of peripheral Th17 cells and serum Th17-related cytokines have been reported in patients with colorectal cancer (124) while a “Th17 expression signature” has been observed in early colorectal cancer (125). It is well-known that IL-1β and IL-6 together with TGF-β can induce differentiation and development of Th17 cells (126, 127). Coincidently, IL-1β and IL-6 also participate in colorectal cancer. Mice deficient in the IL-1 receptor-related molecule SIGIRR show increased tumor growth (128), while tumor-infiltrating myeloid cells produce high levels of IL-1β and IL-6 that promote tumorigenesis (129, 130). IL-6 secreted by lamina propria myeloid cells not only protects IECs from apoptosis but also provides the survival signal for premalignant IECs via the STAT3 pathway (131). Furthermore, treatment with anti-IL-6 in IBD patients has been shown to prevent the onset of CAC (132, 133). Although it is clear that macrophages lacking IL-10 signaling lead to increased development of Th17 cells in severe colitis (77, 79), the molecular mechanisms underlying the complex functions of IL-10 in cancer immune surveillance under inflammatory conditions still remain elusive.

### IL-22

The role of IL-22 in cancer is also complicated. In a healthy condition, IL-22 cannot initiate tumor formation by itself and maintains barrier integrity against cancer development; but under inflammatory conditions, IL-22 directly promotes tumor growth or induces “stemness-like” cancer cells via STAT3-dependent signaling.

In a study on AOM/DSS-induced colorectal cancer, the number and size of tumors were found to increase in IL-22^−/−^ mice compared to WT mice, while the IL-22BP^−/−^ mice were found to exhibit increased tumorigenesis in an NLRP3/NLRP6-IL-18-dependent manner (134), indicating that IL-22 can protect mice against tumor development. Conversely, data also shows that IL-22 results in tumor progression (135, 136). With the elevated IL-17 and IL-22 levels in STAT1^−/−^ mice, the colonic epithelial cell proliferation increases while the tumor apoptosis rate decreases in the early stage of tumor formation (137). In humans, an SNP variation in the human IL-22 gene greatly increases the risk of colon cancer (138). Th22 and IL-22 levels have also been reported to be profoundly increased in CRC patients (139). Moreover, in *Helicobacter pylori*/AOM-induced CRC models, Kirchberger et al. reported accumulation of Nkp46^−^CD4^−^lin^−^Thy1^hi^ ILCs at tumor sites. These ILCs produce IL-22 that promotes cancer development by inducing epithelial cell proliferation through phosphorylation of the Stat3 pathway; neutralization of IL-22 leads to abrogation of this process (140).

STAT3 is oncogenic in colorectal cancer, as evidenced by the observation that mice with specific ablation of STAT3 in IECs develop fewer tumors in colorectal cancer (131, 141). Similarly, overexpression of IL-10Rβ in HT29 promotes IL22/STAT3 signaling in colorectal carcinogenesis (142). Notably, STAT3 can be activated by IL-6, IL-10, IL-11 as well as IL-22 (141). Although the IL-22-STAT3 pathway remains very important for epithelial cell proliferation and CAC development, IL-6- (143) and IL-11- (144) mediated gp130-STAT3 pathway is also required for GI cancer progression.

STAT3 signaling is required not only by the epithelial cells in the tumor microenvironment but also by cancer stem cells. For example, in human colorectal cancer tissues, CCR6^+^CD4^+^ T cells have been shown to be responsible for the secretion of the entire amount of IL-22 (145). IL-22 activates the STAT3 phosphorylation cascade in cancer cells and induces the expression of stem cell markers (SOX2, NANOG, and POU5F1), resulting in increased cancer stemness and tumorigenic potential (146). In line with this, ILC3s also maintain intestinal epithelial stem cells after tissue damage. LGR5 (Leucine-rich repeat containing G protein-coupled receptor) is known to be a stem cell marker in the murine small intestine and colon (147). By using ILC3-deficient Lgr5 reporter mice, Aparicio-Domingo et al. and Lindemans et al. showed that in the absence of ILC3s or IL-22, intestinal stem cells become severely impaired after tissue damage (148), in an STAT3-dependent mechanism (149).

Finally, IL-22 has been shown to directly promote tumor growth by inducing proliferation and exhibiting anti-apoptotic effects on tumor cells in the colon (150) and lung (151). Tumor cells have been found to display a high level of IL-22 receptor expression as well as increased IL-22 production by surrounding tumor infiltrating T cells, indicating that IL-22 promotes tumor proliferation by engaging the STAT3 signaling in tumor tissues. In the AOM/*Helicobacter hepaticus*-induced colorectal cancer model, repair mechanisms for DNA damage potentially lead to the accumulation of mutations. IL-22-driven, iNOS-dependent DNA damage is associated with inflammation and cancer (152).

The diverse roles of IL-22 in cancer immunity are still not clear. It is possible that the generation of IL-22 in different stages of cancer development leads to different consequences. As a potential therapeutic target, more fundamental studies on IL-22 need to be investigated.

## Therapeutic Potential for Il-10 and Il-22 in Intestinal Diseases

Based on the fact that mice deficient in either IL-10 or the IL-10 receptor α or β chains develop spontaneous colitis and multiple anti-inflammatory functions in IBD (60, 119), after proving recombinant IL-10 is safe for human in clinical trial at early 1990s (153), rhuIL-10 was used to treat IBD patients in clinical trial in 2000. In this double-blinded clinical study, IL-10 treatment group shows no significant difference (154). Another clinical study using rhuIL-10 for testing its prevention role for patients with relapse also displays no significant benefit from IL-10 treatment (155). Moreover, increased IFN-γ production (156) and reduced hemoglobin and thrombocyte counts (157) are seen in patients, suggesting a more complex immune function of IL-10 in IBD. It is possible that relative low concentration of delivered rhuIL-10 in the inflamed tissues. To overcome this difficulty, PEGylated IL-10 or IL-10-Fc fusion proteins are designed, respectively (158, 159). Smartly, local delivery of IL-10 by engineered bacterial strains, such as *Lactobacilli* and *bifidobacterial*, have been created to specifically increase IL-10 concentration in the colon (160, 161). *Lactobacilli* and *bifidobacterial* are probiotics which have no apparent capacity to induce mucosal inflammation, preliminary trials about IL-10-engineered probiotics in human IBD patients should be encouraged (162). On the other hand, intravenous IL-10 administration displays no organ specificity (163), it prevents both mucosal and systemic host responses. Therefore, a xylose-inducible expression system (164) has been used to control *Lactobacilli*'s IL-10 production, it leads to a high-level and long-term IL-10 production, which is efficiently delivered to mucosal surfaces (165). Interestingly, the employment of fermented milks as a new form of administration of IL-10-producing *Lactobacilli* is effective in the prevention of mucosal inflammation (166). Despite it, IL-10/IL-10R complex is still an attractive target for cancer immune therapy. Mice treated with CpG plus anti-IL-10Rα have dramatically reduced C26 colon carcinoma growth, while anti-IL-10R or CpG alone does not, indicating blockade IL-10 signaling pathway together with TLR-9 stimulation promotes tumor rejection (167). Recently, PEGylated IL-10 is shown to Induce systemic immune activation, including intra-tumoral CD8^+^ T cells proliferation and expansion, combined PEGylated IL-10 with anti-PD-1 Ab increased the expansion of LAG-3^+^ PD-1^+^ CD8^+^ T cells (168). This result indicates IL-10 can synergize with anti-PD-1 Ab to reverse the dysfunction status of T cells and eliminate the tumor cells. Whether the similar mechanism appears in colorectal cancer need to be explored. Due to the strong immune suppressive functions, IL-10 can repress cytotoxic T cells activation and IL-12 production. But inflammation may promote tissue damage and oncogenesis (169, 170), especially in colorectal cancer. Thereby, IL-10 may inhibit the increased risk of intestinal oncogenesis. The failures of administration of IL-10 in IBD patients stop the step for further treating colorectal cancer patients with IL-10. More knowledge about how inflammation or tissue specific tolerance for tumor proliferation will be helpful to determine using recombinant IL-10 or anti-IL-10R Ab to fight against cancer.

IL-22 plays an essential role in regulating intestinal equilibrium during inflammation. IL-22 not only promotes epithelial cells activation though STAT3 signaling pathway, but also induces various antimicrobial peptides (Claudin-2, and Fut-2) (171), as well as enhances innate intestinal defense functions. IL-22 exerting a beneficial role in various murine colitis models is well-defined (3). As we discussed above, only in some ILC3 cells involved colitis models, such as anti-CD40 and CD45RBl^low^ transferred colitis models, IL-22 plays a pathogenic role (111, 113). Now, IL-22 based clinical trials have been investigated. A recombinant fusion protein, F652, is consisting of two human IL-22 molecules linked to an immunoglobulin constant region (IgG_2_-Fc). F652 was evaluated in a randomized, double-blind, placebo-controlled study. Administration of this hIL-22 dimer to healthy male volunteers is safe and well-tolerated (172). Another hIL-22 IgG fusion protein, UTTR1147A, was assessed in healthy mice, rats and cynomolgus monkeys. Moreover, UTTR1147A is shown to induce STAT3 activation in primary human hepatocytes and human colon cell lines (173). A clinical trial (NCT02847052) for studying the role of IL-22BP in IBD patients is completed, although the results are not available yet. Further clinical trials of F-652 and UTR1147A should be planed for treating inflammatory bowel diseases. Finally, as myeloid cells have higher IL-10 receptor expression while the extensive expression of IL-22R on epithelial cells, a way to induce specific cell lineage responding to these cytokines should be concerned for the future therapeutic method.

## Concluding Remarks

IL-10 and IL-22 are tightly associated with the prevention of mucosal inflammation and have a variety of functions in colorectal cancer development.

IL-10 is necessary for controlling the abnormal immune response against harmful microorganisms and the consequent intestinal damage. The dynamic interactions between IL-10 and the different IL-10 responsive immune cell lineages participate in the pathogenesis of IBD. Apart from Treg cells, macrophages are considered as another major immune subset to respond to IL-10 in the gastrointestinal tract. IL-22 is believed to act exclusively on epithelial cells to promote proliferation and barrier function in the intestine and, therefore, plays a protective role in IBD.

IL-10 leads to tumor growth and promotion, but it also contributes to the eradication and suppression of cancer development under colonic inflammatory conditions. IL-22 is also a controversial cytokine in tumor development; the IL-22-STAT3 axis induces anti-apoptotic genes and provides survival and proliferation signals for both normal and malignant cells. Therefore, in the healthy condition, it prevents tumor formation; however, once a tumor has been established, IL-22 promotes tumorigenesis.

## Author Contributions

H-XW wrote the manuscript. BW and BL provided the guidance and revised the manuscript. All authors contributed to the article and approved the submitted version.

## Conflict of Interest

The authors declare that the research was conducted in the absence of any commercial or financial relationships that could be construed as a potential conflict of interest.
